# High-dose intravenous immunoglobulin therapy for eosinophilic granulomatosis with polyangiitis

**DOI:** 10.1186/2045-7022-4-38

**Published:** 2014-12-12

**Authors:** Naomi Tsurikisawa, Hiroshi Saito, Chiyako Oshikata, Takahiro Tsuburai, Kazuo Akiyama

**Affiliations:** Departments of Allergy and Respirology, National Hospital Organization Sagamihara National Hospital, 18-1 Sakuradai, Minami-ku Sagamihara, Kanagawa, 252-0392 Japan; Clinical Research Center for Allergy and Rheumatology, National Hospital Organization Sagamihara National Hospital, 18-1 Sakuradai, Minami-ku Sagamihara, Kanagawa, 252-0392 Japan

**Keywords:** Eosinophilic granulomatosis with polyangiitis, Churg–Strauss syndrome, Intravenous immunoglobulin, Regulatory T cells, IgG

## Abstract

**Background:**

Regulatory T (T_reg_) cells are implicated in the development and progression of eosinophilic granulomatosis with polyangiitis (EGPA). We previously showed beneficial effects of intravenous immunoglobulin (IVIG) therapy combined with corticosteroid and immunosuppressant treatment on clinical symptoms, including mononeuritis multiplex and cardiac dysfunction, and T_reg_ cell frequency, during EGPA. Whether the timing of administration (during initial treatment or at relapse after remission) or previous treatment affects the clinical and immunologic efficacy of IVIG is unknown. We evaluated whether the frequency of T_reg_ cells varied depending on when IVIG was provided relative to the start of conventional therapy for EGPA.

**Methods:**

The patient population for this retrospective analysis comprised 17 patients with severe mononeuritis multiplex or heart failure whose EGPA did not respond to corticosteroids combined with immunosuppressant therapy. Ten patients first received IVIG during initial treatment, whereas the remaining 7 patients first received IVIG on relapse after remission. We measured the percentage of T_reg_ cells, defined as FOXP3^+^CD4^+^ T cells, present before the first round of IVIG and at 1 month after the last IVIG treatment.

**Results:**

FOXP3^+^CD4^+^ T cells were increased in patients who required only a single course of IVIG to achieve remission compared with those who needed two or more courses. The dosage of prednisolone at initial IVIG was inversely correlated with the ratio of the number of FOXP3^+^CD4^+^ T cells before IVIG and that at 1 month thereafter.

**Conclusion:**

Patients with severe EGPA who receive IVIG after nonresponse to high-dose prednisolone during initial treatment may need multiple courses of IVIG to achieve remission. An increase in the frequency of T_reg_ cells after IVIG may predict the need for additional IVIG in EGPA.

## Background

Eosinophilic granulomatosis with polyangiitis (EGPA; also known as Churg–Strauss syndrome) is a rare disease characterized by allergic granulomatosis and necrotizing vasculitis after peripheral and tissue eosinophilia [1]. The mortality and prognosis of EGPA are related to disease severity, as assessed by five-factor scores (FFSs) [2], and the survival rate at 5 years after myocardial involvement is reported to be one third of that without myocardial involvement [3]. The mainstay of treatment for EGPA is systemic corticosteroid therapy; some patients receive additional treatment with immunosuppressive agents, such as cyclophosphamide and azathioprine [4]. However, combined therapy with corticosteroids and cyclophosphamide afforded little benefit in some EGPA patients with mononeuritis multiplex, heart failure, or systemic vasculitis associated with antineutrophil cytoplasmic autoantibody [5, 6]. We previously showed that intravenous immunoglobulin (IVIG) therapy was effective against severe mononeuritis multiplex or heart failure in patients with EGPA that did not respond to corticosteroid–cyclophosphamide treatment [7].

Regulatory T (T_reg_) cells play a key role in balancing immune responses and in maintaining peripheral tolerance against antigens and allergens [8]. We previously reported a lower frequency of T_reg_ cells at the onset of disease in patients with EGPA than in patients with asthma and that the frequency of T_reg_ cells increased at remission of EGPA [9]. Recent reports show that IVIG regulates the activation of CD4^+^CD25^+^ T_reg_ cells in autoimmune disease [10]. Possible mechanisms involved in the IVIG-mediated modulation of T_reg_ functions include the activation and induction of T_reg_ cells by the Fc region of IVIG and enhancement of T_reg_ function through increased expression of forkhead box P3 (FOXP 3), transforming growth factor-β, interleukin (IL)-10, and cytotoxic T-lymphocyte antigen 4 (CTLA-4) [11–13]; direct interaction of self-reactive natural autoantibodies with T-cell surface molecules [14, 15]; and the suppression of allogeneic T-cell responses through direct activation of T_reg_ cells [16]. We found that prolonged IVIG treatment increased the frequencies of T_reg_ cells, defined as the CD25^+^ subpopulation among CD4^+^ T cells producing IL-10 and the FOXP3^+^ subpopulation among CD4^+^ T cells [17]. Several authors have reported that IVIG treatment may reduce the amount of corticosteroids needed for maintenance of remission of EGPA [17, 18]. However, whether the clinical and immunologic efficacy of IVIG is affected by the timing of administration (e.g., during initial treatment of EGPA or at relapse after remission) is unknown. In addition, the characteristics of patients’ disease condition that influence whether remission is achieved after single or multiple courses of IVIG have not previously been assessed. Here we evaluated whether the frequency of T_reg_ cells differed depending on when IVIG was provided relative to the start of conventional therapy for EGPA.

## Methods

This study was a retrospective analysis for which we recruited patients given IVIG during initial treatment or at relapse after remission, to investigate the clinical and immunologic efficacy of IVIG therapy for EGPA patients.

### Patients

Between March 2005 and November 2011, 17 patients with EGPA were diagnosed according to the classification criteria of the American College of Rheumatology at our hospital [19]. Two of these 17 patients were enrolled in the study reported in reference number 17; the remaining 15 patients were new patients. Multiple mononeuritis, a type of motor nerve dysfunction, was evaluated by using the manual muscle test (MMT); responses were scored (from 0 to 5) on the Medical Research Council scale, and sensory nerve dysfunction was evaluated through clinical examination. Lung involvement was considered to be present when any consolidation, ground-glass opacity, nodules within such opacity, interlobular septal thickening, bronchial wall thickening, lymph node enlargement, pleural effusion evident upon high-resolution computer tomography, or eosinophilic infiltration detected by lung biopsy was present. The heart was considered to be involved when any chest pain, chest discomfort, back pain, palpitations, echocardiographic abnormality, Holter electrocardiographic abnormality, elevation of B-type natriuretic peptide level, or [^123^I]-meta-iodobenzylguanidine myocardial imaging abnormality was evident [20]. Gastrointestinal involvement was indicated by the presence of symptoms of upper or lower abdominal pain, diarrhea, constipation, or positive endoscopic signs combined with confirmation of eosinophilic infiltration by biopsy. Skin involvement was defined as the presence of purpura, erythema, livedo, ulceration, or acrocyanosis when a nodule, accompanied by eosinophilic infiltration, was detected by biopsy. Central nervous system involvement was defined as the presence of headache, visual disorder, abnormal visual sensation, cerebral infarction, bleeding, or cranial nerve dysfunction. Renal involvement was defined by any presence of eosinophils in urine, glomerular nephritis, nephrosis, renal dysfunction (i.e., the creatinine level was 20% or more higher than the baseline figure), or proteinuria (>0.5 g/dL). Disease severity in all EGPA patients was evaluated by using FFS 2009 [2].

All patients treated with IVIG had severe neuropathy (MMT score, <3) or cardiac involvement, including severe heart failure, myocarditis, or pericarditis, which did not improve or achieve remission after at least one month of treatment with conventional therapy (corticosteroids, immunosuppressants, or both; the initial dose of corticosteroid was approximately 1 mg/kg prednisolone daily for at least 1 month) [4]. Among our 17 patients with EPGA, 10 received IVIG during initial treatment with conventional therapy; the remaining 7 patients achieved first remission after conventional treatment without IVIG and therefore received their first IVIG on relapse after remission. Remission was defined as the absence of any clinical signs or symptoms of active vasculitis for at least 6 months. Relapse was defined as the presence of active disease combined with the recurrence, after initial remission, of vasculitis symptoms (with or without an increase in the percentage of eosinophils among white blood cells [WBCs]). Patients who relapsed required resumption of immunosuppressive therapy or increased doses of immunosuppressant.

### Study design

Before treatment with IVIG (Venilon, Teijin, Tokyo, Japan; 400 mg/kg daily for 5 days), all patients received corticosteroids (initial dose, about 1 mg/kg prednisolone daily for at least 1 month) with or without immunosuppressants. Nine patients with treatment-refractory mononeuritis multiplex or heart failure received additional courses of IVIG at 2 to 12 months after the initial IVIG therapy until they achieved remission. As in our previous study [17], we measured the percentage of CD25^+^CD4^+^ T_reg_ cells (that is, CD25^+^ cells among CD4^+^ T_reg_ cells) and FOXP3^+^CD4^+^ T cells (that is, FOXP3^+^ cells among CD4^+^ T cells), which we defined as T_reg_ cells, in the peripheral blood of all patients before and at 1 month after the initial IVIG treatment; the ratio between these values was used to represent the IVIG-associated change in the proportion of FOXP3^+^CD4^+^ T cells. The ethics committee of our hospital approved the study, and we obtained informed consent from each patient.

### Immunologic analysis

The number of eosinophils in whole blood before and at the onset of IVIG therapy, and the serum IgG concentration before IVIG, were measured in all patients. Eosinophils and WBCs in the peripheral blood were counted by hemocytometry. The frequencies of CD25^+^CD4^+^ T cells and of FOXP3^+^CD4^+^ T cells were determined as described by Abdulahad et al. [21]. PE-conjugated anti-human FOXP3 and Peridinin chlorophyll conjugates of mouse IgG1 and an anti-CD4 antibody were purchased from BD Biosciences (Rikaken Co. Ltd., Tokyo, Japan). In brief, whole-blood lymphocytes were incubated with FITC-conjugated anti-CD4, and FOXP3^+^CD4^+^ T cells (defined as FOXP3^+^ cells among CD4^+^ T cells) were identified by incubating whole-blood lymphocytes with PE-conjugated anti-FOXP3 antibody after cell permeabilization with 4% (v/v) formaldehyde and 0.1% (w/v) saponin. The expression of surface and intracellular markers by CD4^+^ T cells was analyzed by means of flow cytometry (FACSCalibur, Nippon Becton Dickinson, Tokyo, Japan).

### Statistical analysis

All values are expressed as mean ± 1 SD unless otherwise specified. Statistical comparisons among groups were achieved by using two-way analysis of variance (ANOVA) according to a repeated-measures algorithm, followed by *post-hoc* comparisons by using the Newman–Keuls test. The two mean values obtained by this process were compared by using the Wilcoxon matched-pairs *t* test. Correlation coefficients were obtained by using Spearman’s rank correlation test. *P* values less than 0.05 were considered statistically significant. Statistical analysis was performed by using SPSS for Windows, version 20 (SPSS Inc., Chicago, IL).

## Results

A single course of IVIG after conventional corticosteroid-based therapy was sufficient to achieve remission of EGPA in 3 of the 10 patients who received IVIG during their initial treatment and in 5 of the 7 patients treated with IVIG after relapse of EGPA. The other nine patients required 3.6 ± 1.6 course of IVIG before remission was achieved. Overall, 8 patients that received a single round of IVIG treatment during initial therapy to achieve remission, and the remaining 9 patients needed two or more IVIG treatments after relapse to achieve a second remission. Although the dosage of prednisolone at initial treatment did not differ between the two groups, the proportion of patients who received immunosuppressants was greater (*P* < 0.05) for those given IVIG during initial therapy than for those treated with IVIG after relapse of EGPA (Table 1). The dosage of prednisolone at initial IVIG in patients with relapsed disease was lower (*P* < 0.05) than that for patients under initial treatment for EGPA. Whereas 9 of the 10 patients (90.0%) who received IVIG during their initial treatment received immunosuppressants in addition to corticosteroids, only 3 of the 7 patients (42.9%) with relapsed EGPA received immunosuppressants during their initial treatment (*P* < 0.05) (Table 1).

**Table 1:** **Patient characteristics**

	EGPA patients who received IVIG during initial treatment (***n*** = 10)	EGPA patients who received IVIG on relapse after remission (***n*** = 7)	***P***
Age (y), mean ± 1 SD	59.1 ± 15.2	47.9 ± 18.3	NS^†^
Sex (M/F)	4/6	3/4	NS^*^
Age at onset EGPA (y), mean ± 1 SD	57.0 ± 42.6	42.6 ± 20.0	NS^†^
At onset of EGPA			
WBC (/μL), mean ± 1 SD	13,573 ± 4,647	16,930 ± 6,728	NS^†^
Blood eosinophils (/μL), mean ± 1 SD	6,681 ± 3,698	7,363 ± 6,686	NS^†^
MPO-ANCA (%) at onset	40	28.6	NS^*^
PR3-ANCA (%) at onset	0	0	NS^*^
At initial IVIG treatment			
WBC (/mL), mean ± 1 SD	9,912 ± 3,339	8,093 ± 2,752	NS^†^
Blood eosinophils (/μL), mean ± 1 SD	79.6 ± 69.2	252.3 ± 182.7	<0.05^†^
FOXP3^+^CD4^+^ T cells (%), mean ± 1 SD	2.4 ± 1.6	1.3 ± 1.1	<0.05^†^
Time from onset of EGPA to initial IVIG treatment (mo), median (range)	3.0 (1–3)	65.0 (10–122)	<0.01^††^
Number of IVIG treatments needed to achieve first remission (one/two or more)	3/7	5/2	NS^*^
Initial treatments at onset			
Prednisolone (mg), mean ± 1 SD	50.5 ± 10.5	48.6 ± 10.7	NS^†^
Patients taking an immunosuppressant (%)	90	42.9	< 0.05^*^
CYC/AZA/CSA	8/0/1	1/1/1	NS^*^
Other treatments at initial IVIG			
Prednisolone (mg), mean ± 1 SD	39.5 ± 11.2	12.9 ± 5.5	0.01^†^
Patients taking an immunosuppressant (%)	90	71.4	NS^*^
CYC/AZA/CSA	8/0/1	1/3/1	0.05^*^

AZA, azathioprine; CYC, cyclophosphamide; CSA, cyclosporin; EGPA, eosinophilic granulomatosis with polyangiitis; IVIG, intravenous immunoglobulin; MPO-ANCA, myeloperoxidase-specific antineutrophil cytoplasmic antibodies; NS, not significant; PR3, protein 3; WBC, white blood cells.

All values are expressed as means ± 1 SD.

Values of *P* < 0.05 were considered statistically significant.

^†^Two-way ANOVA with repeated measures between groups.

^††^Statistical comparisons made by using Mann–Whitney U-tests between groups.

*Chi-squared testing revealed no significant differences in frequencies between the two groups.

The number of eosinophils in peripheral blood at initial IVIG was higher (*P* < 0.05) and the percentage of FOXP3^+^CD4^+^ T cells was lower (*P* < 0.05) in patients with relapsed EGPA than in patients undergoing initial treatment (Table 1). The minimal MMT score at onset of treatment was lower (*P* = 0.05) in patients undergoing initial treatment than in patients treated for relapsed EGPA. The percentage of patients with myocardial involvement at initial diagnosis of EGPA was higher (*P* = 0.05) in those treated because of relapsed disease than in those who received their initial treatment for EGPA (Table 2). The diagnoses according to the criteria of the American College of Rheumatology are shown in Table 3.

**Table 2:** **Organ involvement at onset (%)**

	EGPA patients treated with IVIG within the period of initial treatment (***n*** = 10)	EGPA patients treated with IVIG on relapse after remission (***n*** = 7)	***P***
Asthma	100	100	NS^*^
Paranasal sinusitis	90	85.7	NS^*^
Multiple polyneuropathy	100	100	NS^*^
Minimum MMT score, mean ± 1 SD	3.1 ± 0.9	4.1 ± 0.7	0.05^†^
Pulmonary infiltrates	80	85.7	NS^*^
Myocardial involvement	50	100	0.05^*^
Gastrointestinal tract	80	100	NS^*^
Liver, gall bladder, pancreas	16.7	30	NS^*^
Renal involvement^‡^	20	28.5	NS^*^
Proteinuria	50	42.8	NS^*^
Eosinophils in urine	28.6	40	NS^*^
Nephritis or nephrosis	10	14.3	NS^*^
Skin involvement	90	100	NS^*^
Arthritis	40	42.8	NS^*^
Myalgia	40	28.5	NS^*^
Central nervous system involvement	30	28.5	NS^*^
Number of organs involved per patient^‡‡^, mean ± 1 SD	5.7 ± 1.1	6.3 ± 2.1	NS^†^
FFS2009	1.5 ± 1.0	1.4 ± 1.0	NS^†^

EGPA, eosinophilic granulomatosis with polyangiitis; FFS, five-factor score; IVIG, intravenous immunoglobulin; MMT, manual muscle test; NS, not significant.

All values are expressed as means ± 1 SD.

Values of *P* ≤ 0.05 were considered statistically significant.

^†^Two-way ANOVA with repeated measures between groups.

*Chi-squared testing revealed no significant differences between groups.

^‡^Renal involvement including proteinuria or eosinophils in urine or glomerular nephritis or nephrosis or renal dysfunction.

^‡‡^Cumulative organ involvement excluding asthma and sinusitis.

**Table 3:** **Diagnosis by ACR criteria at onset**

	EGPA patients who received IVIG during initial treatment (***n*** = 10)	EGPA patients who received IVIG on relapse after remission (***n*** = 7)	***P***
Asthma (yes/no)	10/0	7/0	NS^*^
Paranasal sinusitis (yes/no)	9/1	6/1	NS^*^
Multiple polyneuropathy (yes/no)	10/0	7/0	NS^*^
Pulmonary infiltrates (yes/no)	8/2	6/1	NS^*^
Extravascular eosinophils (pathology) (yes/no)	10/0	7/0	NS^*^
Eosinophilia in peripheral blood >10% (yes/no)	10/0	7/0	NS^*^
ACR 5/6 (%)	30	57.1	
ACR 6/6 (%)	70	42.9	

ACR, American College of Rheumatology, EGPA, eosinophilic granulomatosis with polyangiitis; IVIG, intravenous immunoglobulin; NS, not significant.

All values are expressed as means ± 1 SD.

Values of *P* < 0.05 were considered statistically significant.

*: Chi-squared testing revealed no significant differences between the two groups.

**Figure 1:**
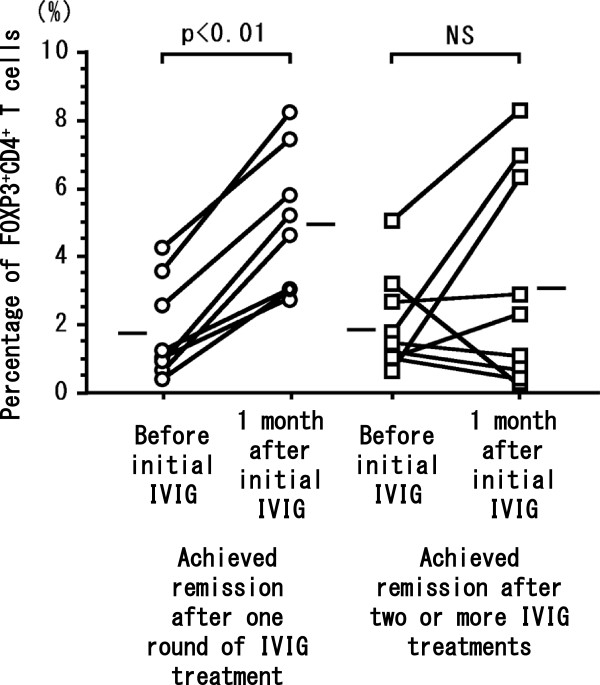
**The percentage (mean ± 1 SD) of FOXP3** ^**+**^ **CD4** ^**+**^ **T cells in 17 EGPA patients just prior to the initial intravenous immunoglobulin (IVIG) treatment and 1 month thereafter.** Open circles represent patients with EGPA (*n* = 8) who achieved remission after a single round of IVIG treatment; open squares represent patients with EGPA (*n* = 9) that required multiple courses of IVIG to achieve remission. The mean values for each patient group were compared by using the Wilcoxon matched-pairs *t*-test; *P* < 0.05 was considered statistically significant. NS, not significant.

**Figure 2:**
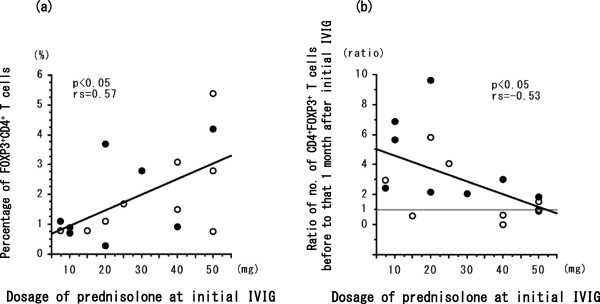
**Statistical correlation between the dose of prednisolone given to patients during initial IVIG treatment and (a) the percentage of FOXP3** ^**+**^ **CD4** ^**+**^ **T cells or (b) the ratio of the percentage of FOXP3** ^**+**^ **CD4** ^**+**^ **T cells before the initial IVIG treatment to that at 1 month thereafter.** Open circles represent patients with EGPA (*n* = 9) who needed two or more IVIG treatments to achieve remission; closed circles represent patients with EGPA (*n* = 8) who achieved remission after a single course of IVIG. Correlation coefficients (rs) were obtained by using Spearman’s rank correlation test.

**Figure 3:**
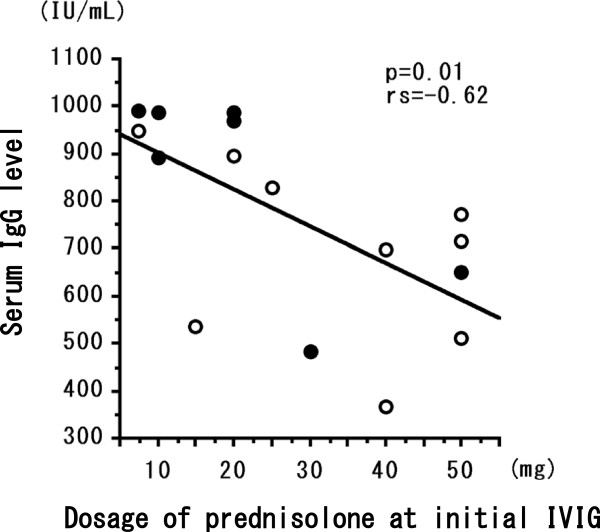
**Statistical correlation between the dose of prednisolone during the initial IVIG treatment and the serum IgG level in 16 EGPA patients (one patient did not measure serum IgG).** Open circles represent patients with EGPA (*n* = 9) who needed two or more IVIG treatments to achieve remission; closed circles represent patients with EGPA (*n* = 7) who achieved remission after a single course of IVIG. Correlation coefficients (rs) were obtained by using Spearman’s rank correlation test.

**Figure 4:**
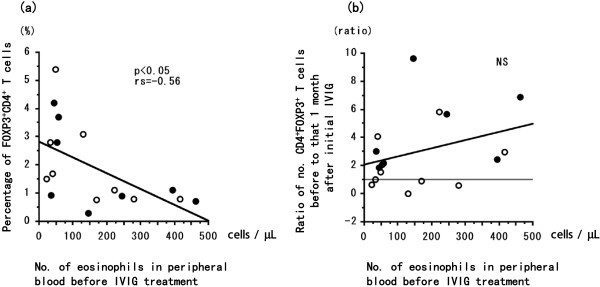
**Statistical correlation between the number of eosinophils in the peripheral blood before IVIG treatment and (a) the percentage of FOXP3** ^**+**^ **CD4** ^**+**^ **T cells or (b) the ratio of the percentage of FOXP3** ^**+**^ **CD4** ^**+**^ **T cells before the initial IVIG treatment to that at 1 month thereafter.** Open circles represent patients with EGPA (*n* = 9) who needed two or more courses of IVIG to achieve remission; closed circles represent patients with EGPA (*n* = 8) who achieved remission after a single round of IVIG. Correlation coefficients (r) were obtained by using Spearman’s rank correlation test.

The percentage of FOXP3^+^CD4^+^ T cells at initial IVIG was significantly (*P* < 0.05) greater in the 8 patients who achieved remission after a single round of IVIG compared with the 9 patients who needed two or more IVIG treatments for remission of EGPA (Figure 1). In all patients, the dosage of prednisolone at initial IVIG was significantly statistically correlated (*P* < 0.05, rs = 0.57) with percentage of FOXP3^+^CD4^+^ T cells (Figure 2a) and significantly inversely correlated (*P* < 0.05, rs = −0.53) with the ratio between the number of FOXP3^+^CD4^+^ T cells before to that at 1 month after the initial IVIG treatment (Figure 2b). The percentage of FOXP3^+^CD4^+^ T cells before the initial course of IVIG was greater than that at 1 month afterward in 5 of the 9 patients who needed two or more rounds of IVIG to achieve remission of EGPA (Figure 2b). In all patients, serum IgG concentration (IU/mL) before initial IVIG was significantly inversely correlated (*P* < 0.01, rs = −0.62) with the dosage of prednisolone at initial IVIG (Figure 3) but was not correlated with duration or the total dose of prednisolone taken during this study. The number of eosinophils in peripheral blood before IVIG was significantly inversely correlated (*P* < 0.05, rs = −0.56) with the percentage of FOXP3^+^CD4^+^ T cells (Figure 4a) but not with the ratio of FOXP3^+^CD4^+^ T cells before to that 1 month after initial IVIG (Figure 4b).

## Discussion

Treatment with IVIG has shown efficacy in autoimmune and immune-mediated inflammatory diseases [14, 15, 22], including microscopic polyangiitis, granulomatosis with polyangiitis (that is, Wegener’s granulomatosis), and antineutrophil cytoplasmic antibody-associated systemic vasculitis [5, 23, 24]. The phase at which IVIG is most effective in EGPA patients with neuropathy or cardiac failure is unknown, as are the optimal number and timing of courses of IVIG to induce remission. Among patients with systemic lupus erythematosis, responders and nonresponders to IVIG treatment differed in their serum concentrations of C4, SS-A, and SS-B [22]. We previously reported that the number of courses of IVIG needed to induce remission was 2.6 ± 2.1 times greater to achieve first remission [17]. Whether differences in disease characteristics affect the need for one compared with multiple rounds of IVIG treatment to induce remission is unknown. We here show that patients with severe EGPA that receive IVIG during their initial treatment with high-dose systemic corticosteroids likely need multiple courses of IVIG to achieve remission. In addition, an increased percentage of FOXP3^+^CD4^+^ T cells before IVIG treatment may indicate an increased likelihood of induction of remission of EGPA. In patients receiving their initial treatment for EGPA, the percentage of FOXP3^+^CD4^+^ T cells was increased after conventional treatment comprising corticosteroids with or without immunosuppressants. However, the percentage of FOXP3^+^CD4^+^ T cells at one month after IVIG failed to increase in more than half of the patients given prednisolone doses exceeding 40 mg during IVIG treatment (Figure 2b), and high-dose prednisolone may suppress the serum IgG level (Figure 3). We consider that many inflammatory mediators are present in various quantities in serum during the acute disease phase associated with initial treatment of EGPA; these mediators might therefore out-compete IVIG and suppress the differentiation of T_reg_ (FOXP3^+^CD4^+^) cells.

The number of T_reg_ cells (i.e., FOXP3^+^CD4^+^ T cells) in patients with severe EGPA did not increase rapidly after IVIG, and this cell population was increased in patients requiring multiple courses of IVIG to achieve clinical remission (data not shown). We previously reported that the frequency of CD25^+^CD4^+^ T cells correlated with FOXP3^+^CD4^+^ T_reg_ cells in EGPA patients with severe mononeuritis multiplex or cardiac dysfunction and remained greater than that in EGPA patients without IVIG treatment for as long as 2 years after completion of IVIG therapy [17].

EGPA manifests as necrotizing vasculitis and eosinophilic tissue inflammation [25, 26]. In both of our current patient groups, the number of eosinophils in peripheral blood decreased after conventional treatment and was significantly inversely correlated with the percentage of FOXP3^+^CD4^+^ T cells. However, the number of eosinophils at initiation of IVIG treatment was unrelated to any increase in the percentage of FOXP3^+^CD4^+^ T cells after IVIG. These results indicate that disease activity and response to IVIG likely are reflected not only in the number of peripheral eosinophils but also in levels of Th2 cytokines [27–29], Th17 [27, 28, 30, 31], or other unknown mediators. Other possible mechanisms involved in IVIG-mediated inhibition are the differentiation and function of Th17 cells [32], the upregulation of T_reg_ cells by induction of cyclooxygenase-2-dependent prostaglandin E2 [33], or the suppression of inflammation by upregulating FcγRIIB on macrophages via IL-4 produced by basophils [34]. Although *in vitro* experiments have hinted at many novel findings regarding the mechanisms of IVIG, it is necessary to verify the clinical or immunologic effects of IVIG *in vivo*. It is useful clinically to determine optimal dose level, timing of initial dose, number of courses, and interdose interval for maximally effective IVIG therapy.

## Conclusion

We conclude that most patients with otherwise uncomplicated non-severe EGPA (i.e., MMT score greater than 3, lack of cardiac involvement) who receive IVIG at relapse can achieve remission after a single round of IVIG therapy. However, many patients with severe EGPA (i.e., MMT score less than 3, cardiac abnormalities) who are undergoing initial treatment with conventional therapy including high-dose prednisolone will require multiple courses of IVIG to achieve remission of EGPA.

## Abbreviations

CTLA-4: Cytotoxic T-lymphocyte antigen 4
EGPA: Eosinophilic granulomatosis with polyangiitis
FFS: Five-factor score
FOXP3: Forkhead box P3
IL: Interleukin
IVIG: Intravenous immunoglobulin
Treg cells: Regulatory T cells
MMT: Manual muscle test
MPO-ANCA: Myeloperoxidase-specific antineutrophil cytoplasmic autoantibody
WBC: White blood cells.
